# Prevalence of Traditional Medicine Use during Pregnancy, at Labour and for Postpartum Care in a Rural Area in Zimbabwe

**DOI:** 10.24105/2090-7214.16.321

**Published:** 2019-04-30

**Authors:** Tariro Mawoza, Charles Nhachi, Thulani Magwali

**Affiliations:** 1 Department of Clinical Pharmacology, University of Zimbabwe, Harare, Zimbabwe; 2 Department of Obstetrics and Gynaecology, University of Zimbabwe, Harare, Zimbabwe

**Keywords:** Pregnancy, *Fadogia ancylantha*, Elephant dung, Prevalence, Traditional medicine

## Abstract

**Objectives:**

The aim of this study was to determine the prevalence and types of traditional medicine used during pregnancy, at labour and for postpartum care by women in rural Zimbabwe.

**Research design:**

A cross-sectional survey was conducted on 398 women from two rural districts in Zimbabwe. Data on socio-demography, pregnancy related information as well as traditional medicine use patterns was collected using a structured interviewer administered questionnaire. Convenient sampling was used to recruit women of childbearing age who were either pregnant at the time of the study, or had previously given birth.

**Results:**

The prevalence of traditional medicine used during pregnancy and at labour was 69.9% and only 17.3% used these medicines for postpartum care. During pregnancy, 27.7% used soil from a mole hill, 21.6% used elephant dung, and 13.3% used *Fadogia ancylantha.* These medicines were mainly used to facilitate labour (43.5%), avoid tears/stitches (19.7%), make delivery easy and safe (18.3%) and to avoid prolonged labour (5%). Only 9% of the participants however reported to have experienced adverse effects from using traditional medicines.

**Conclusion:**

The use of traditional remedies in different forms during pregnancy and at labour was very common as confirmed by the high prevalence rate of 69.9%. Some of the women however used more than one type of traditional medicine during pregnancy, labour and for postpartum care. The exact effects of some of these medicines on both the mother and infant however, are not known, and there is therefore a need for them to be studied in greater detail.

## INTRODUCTION

The Centre for Gender and Social Policy Studies [1] estimates that 85% of the population in developing countries depends mainly on traditional healthcare systems. The World Health Organization (WHO) however, estimates that 60% of the world’s population depends on traditional medicines; with up to 80% of the population in Africa using traditional medicines to meet their daily healthcare needs [2]. A study by Robinson [3] estimated that these rates could be as high as 95%.

In some African communities, traditional medicines have been used by women during pregnancy and at childbirth with reportedly high prevalence rates. In Ethiopia for example, a study determining prevalence of herbal medicine use showed that 73.1% (285) of the women interviewed used traditional medicines during pregnancy [4]. Similarly, studies conducted in an urban area in Zimbabwe showed the prevalence of prenatal and antenatal use of traditional medicines to be 52%, suggesting high use [5]. In a Ugandan study however, the prevalence rates were reported to be lower at 20% [6], whilst in Kenya, only 12% of the women who had access to public healthcare used traditional medicines during pregnancy [7]. In a multinational study comparing the use of traditional medicines in Europe, Russia had the highest prevalence of herbal medicine use in pregnancy at 69%, whilst Sweden had the lowest at 4.3% [8]. Australian prevalence however, ranged between 11% and 56% [8].

These varying prevalence rates suggest that women use traditional medicines during pregnancy, for many reasons [9]. This is mainly because: the information on their use has been passed down from generation to generation, they are perceived to be safer and more efficacious than conventional medicines in pregnancy, and they are readily available [6]. Therefore with the prevalence of traditional medicine use in pregnancy being estimated to be between 12 and 45% in many countries, the World Health Organization has been encouraging the development of structures that support the integration of traditional medicines into health care systems [10]. This mainly involves the production of guidelines on their proper use through the stimulation of strategic research into traditional medicines by providing support for clinical research projects on their safety and effectiveness [10].

Although traditional medicine use in pregnancy is reportedly also common in Zimbabwe, information regarding some of the commonly used medicines is lacking. This study was thus aimed at determining the traditional medicines that are currently being used by women in rural Zimbabwe during pregnancy, labour and for postpartum care, determining the pattern of use, as well as sources of information and supply of these medicines. The results obtained from this study will allow for information regarding the commonly used medicines to be passed on to health care practitioners to ensure that women receive adequate treatment during pregnancy or at delivery. In addition, study results can be used as a guide for further detailed pharmacological studies of these medicines.

## MATERIALS AND METHODS

### Methodology

A cross sectional survey was conducted using an interviewer administered questionnaire to interview eligible women who visited clinics in the *Goromonzi* (17.8108° S, 31.3542° E) and Murehwa (17.6452° S, 31.7822° E) Districts in Zimbabwe. The questionnaire used contained a mixture of open and close ended questions.

### Ethical considerations

Ethical approval for the study was obtained from the Joint Research Ethics Committee for the University of Zimbabwe, College of Health Sciences and Parirenyatwa hospitals as well as the Medical Research Council of Zimbabwe. Written informed consent was obtained from all the study participants.

Permission to conduct the study in the two districts was granted by the Provincial Medical Director for Mashonaland East and the Goromonzi District Medical Officer.

### Study population, inclusion criteria for participants and sample size calculation

A convenient sample of women who visited the local clinics in the two districts was enrolled into the study. Eligible women were within the child bearing age group (18–49 years old), and either pregnant at the time the study was being conducted or women who had been pregnant and/or given birth before. Study participants had to be able to give written informed consent. We calculated a sample size of 384 using Dobson formula [11].

### Data collection technique

A structured interviewer administered questionnaire, in the preferred language of the participant (English or Shona), was used to collect data. Women were recruited daily until required numbers were reached. Data were collected from May 2017 to July 2017.

The questionnaire collected the following information:

- Identifying the traditional medicines that were/are used by women during pregnancy, at labour and for postpartum care
- Sources of information and supply of these traditional medicines
- The outcomes that are associated with use of traditional medicines during pregnancy, at labour and for postpartum care
- Any adverse effects/complications associated with using traditional medicines
- Socio-demographic characteristics of the participants

### Pilot study

A pilot study was conducted at the Show grounds clinic in Domboshava (Goromonzi district) with 20 participants. The average time taken to complete the questionnaire was 5 minutes, with corrections being noted. Editions to the original questionnaire were then made.

### Data processing and analysis

Collected data were entered into RedCAP© and Epi-info and SPSS was used to analyse data. Data were de-identified for confidentiality and the anonymity of the participants was guaranteed through the use of study-generated participant codes.

## RESULTS

### Demographic details

Of the 412 women who were approached, 398 women agreed to participate in the study. These women were conveniently recruited from the two districts from May 2017 to August 2017. 81% (n=323) of the study participants were married, and women in the 21–40 year age group had the highest number of women who had used traditional medicines during pregnancy and at birth.

The rest of the characteristics of the study participants are summarized in Table 1 along with their respective p-values.

### Use of traditional medicines during pregnancy and at childbirth

Of the study participants only 9% reported to using traditional medicine in an effort to conceive. Two hundred and seventy-eight (69.9%) women however reported to have used traditional medicine during pregnancy and at childbirth. Soil from a mole hill, commonly called ‘Ivhu renhuta’ was used by 77 (27.7%) of the women, while Elephant dung was used by 60 (21.6%) and *Fadogia ancylantha* was used by 13.3% of the women (Table 2). Only 2.9% of the women reported to use the Muddy nest of a Mud dauber at childbirth.

Only 69 (17.3%) women however, reported using traditional medicine for postpartum care. The medicines they used were mainly to reduce the size of the vagina, reduce bleeding and to encourage lactation. These included: *Elephantorrhiza elephantina, Sclerocarya birrea,* and lemons which were used either in combination or as single agents.

### Factors influencing the decision to use traditional medicines during pregnancy and at childbirth

194 (48.7%) women reported that the use of traditional medicines during pregnancy, birth and for postpartum care was good. The reasons highlighted for using traditional medicines included: to facilitate childbirth (n=121, 43.5%), to avoid tears or stitches (n=55, 19.7%), to make delivery safe and easy (n=51, 18.3%), and to avoid prolonged labour (n=14, 5%). Other reasons such as ease of access and the fact that traditional medicines were part of their culture were reported by 21 (7.6%) of the women. However, most of these women used these medicines for multiple reasons.

131 (33%) out of the 398 women reported that the use of traditional medicines in pregnancy had harmful effects, with 89 (22.4%) being non-traditional medicine users and 42 (10.6%) being users. 18.3% of the study participants were however, indifferent/unsure of their effects. Those against the use of traditional medicines believed that the medicine may affect the health of the mother and baby as well as increasing the risk of cancer.

### Sources of information and supply of traditional medicines

**Figure 1:**
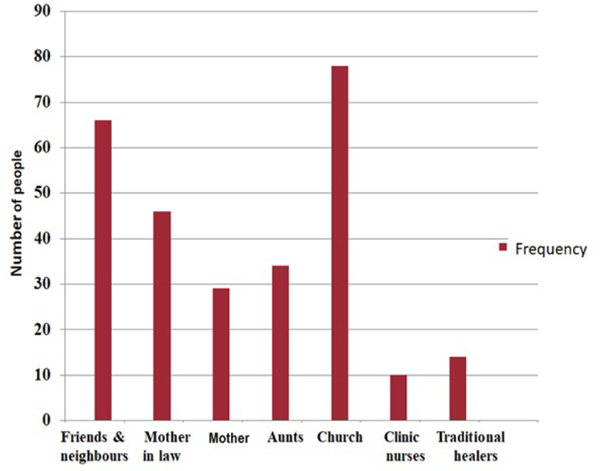
Sources of information and supply of traditional medicines during pregnancy and at delivery.

Of the 278 women who reported to using traditional medicines during pregnancy and at childbirth, 66 (23.7%) reported to have received information as well as supply from friends and relatives, while 78 (28.1%) received it from the Apostolic Faith church (holy water). Only 14 (5%) received the information/supply from traditional healers (Figure 1).

### Adverse events profile

Amongst the women who reported to using traditional medicine during pregnancy and at childbirth, only 26 (9.4%) reported to have experienced adverse effects (Table 3). The most common reported adverse effects were itchiness (7, 27%) and that the medicines left a bitter taste in the mouth (4, 15.4%).

## DISCUSSION

The use of traditional medicines (TM) has been reported to be a common practice in both developed and developing countries [12]. This is mainly because of the long history associated with their use in these countries [12]. The present study was aimed at determining the prevalence of traditional medicine use and determining the types of medicines that are used during pregnancy, at labour and for postpartum care by women in a rural area in Zimbabwe.

A prevalence rate of 69.9% use of traditional medicines during pregnancy and at delivery was reported by the women who participated in this study. No association was noted however between the use of these traditional medicines and any of the demographic characteristics. A similar study conducted in an urban area in Zimbabwe however showed use during pregnancy to have a prevalence of 52% [13]. In their study, Mureyi et al. [5] noted that the use of traditional medicines in pregnancy was associated with: the 20–25 age group nulliparity, nulligravidity, and residing in the Mbare high density neighbourhood. Surprisingly, no association was noted between employment status or education level and the use of traditional medicine. One would have expected that being educated or employed would have had an effect on ones willingness to use traditional medicines.

The differences in prevalence rates between the two studies could however, be attributed to the differences in study location. People in rural areas rely on TM to meet their primary health care needs mainly because these medicines are easily available and are usually free. Primary health care centres however, are not usually accessible due to their location and the inability of rural folk to pay for services. In the urban areas however, there are numerous primary health care centres, which could explain why lower prevalence rates of traditional medicine use were reported in urban areas. The observed high prevalence of TM use in pregnancy and during labour in both study settings, could however be explained by the fact that Zimbabwean culture and traditions encourage pregnant women to use traditional medicines to either treat pregnancy related illnesses or to facilitate labour as they are believed to be safe [5].

When compared with other countries such as western Ethiopia, Malaysia and Kenya whose prevalence rates were 50.4%, 51.4% and 12%, respectively, [7,13,14], TM use during pregnancy and at delivery in Zimbabwe was high. These differences could be associated with differences in culture, ease of accessibility as well as affordability of the medicines. In this study, most of the medicines that women used where either free (from the homestead or the forest) or cost less than US$1. In Zimbabwe, conventional medicines are expensive and the average household income wage is $253 a month (for the 30% of the population who are employed) [15]. This therefore makes it difficult for the average Zimbabwean to access conventional medicines suggesting a willingness to use more affordable medicines [16], which could explain the results obtained in this study.

Although study participants gave varying reasons for using traditional medicines during pregnancy and at labour, most (43.5%) of them believed that traditional remedies facilitate labour. Study findings are comparable with data from a study by Azriani et al. [17] in Malaysia. In their study, Azriani et al. [17] observed that 108 (51.4%) women out of 210 used at least one type of herbal medicine during pregnancy with the most common indication being to facilitate labour (89.8%). Other indications included to avoid tears/stitches, make delivery safe and easy, and to avoid prolonged labour, suggesting the presence of multiple claimed therapeutic benefits of TM use during gestation. Unpublished studies conducted in our laboratory confirmed that some of these traditional medicines (aqueous extracts of Elephant dung and Muddy nest of a mud dauber) contract the uterus, suggesting they may actually facilitate labour and delivery [18].

Of the traditional medicines used, soil from a mole hill was the most commonly used agent (27.7%) followed by Elephant dung (21.6%). 31.3% of the study participants however used different herbal/plant based medicines during pregnancy and at delivery. These results are comparable with those from a study by Tabatabace [19] in Iran whereby 30.8% of the participants had used herbal drugs during pregnancy. Similarly, 36% of the women in an Australian study used at least one herbal supplement during pregnancy [20]. The pharmacological effects of some of the agents used by Zimbabwean women in pregnancy, however, have not yet been scientifically evaluated. In addition, other than the women who had obtained the medicines they were using from the Apostolic Faith church (28.1%), most (23.7%) of the study participants reported to have obtained the information from friends and neighbours. Only 5% however, received traditional medicine information/supply from traditional healers. These results suggest that family members and friends play a huge role in influencing the use of traditional medicine during pregnancy. This has mainly been attributed to cultural practices which have been passed down from generation to generation.

Traditional knowledge and the use of plant-based medicines remains an important aspect in many African countries, especially Zimbabwe. The main challenge with using these medicines however still remains the unavailability of supporting scientific information. Studies by various authors have shown that some of the plants used are toxic if used in large doses. A good example is plants that are used in pregnancy yet they have been reported to be toxic are: *Ricinus communis* and some Aloe vera species. The seeds from *Ricinus communis* which are used in the production of castor oil have been associated with intoxication effects *via* the effects of ricin which is found in these seeds [21]. In addition, oral consumption of some Aloe vera plants shows that the plants possess potential toxic and carcinogenic effects since it contains various polysaccharides and phenolic chemicals, notably anthraquinones [21]. Aloe preparations are therefore associated with diarrhoea, hypokalemia, pseudomelanosis coli, kidney failure, as well as phototoxicity and hypersensitive reactions [22]. Caution should therefore be practiced when some of these plants are being used.

## CONCLUSION

The use of traditional remedies during pregnancy and at labour in the study population was extensive as confirmed by the high prevalence rate of 69.9%. A number of traditional medicines were reportedly used by the study participants during pregnancy, at labour and for postpartum care with the most commonly used TM being: soil from a mole hill, elephant dung, holy water from the apostolic sect church and *Fadogia ancylantha.* Some of the study participants also reported that they used more than one traditional medicine at a time. The challenge with such practices is that it might have deleterious effects on both the mother and the unborn foetus. It is therefore imperative that more studies are conducted so as to determine the pharmacological and toxicological effects of these traditional medicines, *in vitro* and *in vivo.* These studies could involve determining *in vitro* and *in vivo* effects of these medicines using animal models, as well as hepatic and nephrotoxic effects.

## LIMITATIONS

Data collection was based on what the interviewees reported which could introduce an element of recall bias. In addition, the study was conducted in selected rural areas which might not have been a true representation of Zimbabwe as a whole which might have limited the generalizability of the results.

## Figures and Tables

**Table 1:** Demographic details of the study participants.

Demographic characteristic	Total	Used traditional medicine in pregnancy		p-value

		YES (N, %)	NO (N, %)

Age group				0.104

18–20 years	47	35 (74.5)	12 (25.5)	

21–30 years	185	124 (67.0)	61 (33.0)	

31–40 years	113	87 (77.0)	26 (23.0)	

41–49 years	53	32 (60.4)	21 (39.6)	

Marital status				0.759

Single	13	10 (76.9)	3 (23.1)	

Married	323	227 (70.3)	96 (29.7)	

Widowed	23	14 (60.9)	9 (39.1)	

Divorced	20	15 (75.0)	5 (25.0)	

Cohabiting	19	12 (63.2)	7 (36.8)	

Level of education				0.183

No education	6	3 (50.0)	3 (50.0)	

Primary	80	51 (63.8)	29 (36.3)	

Secondary	302	215 (71.2)	87 (28.8)	

Tertiary	10	9 (90.0)	1 (10.0)	

Religion				0.866

Christian	371	259 (69.8)	112 (30.2)	

Muslim	9	6 (66.7)	3 (33.3)	

African tradition	10	8 (80.0)	2 (20.0)	

Other	8	5 (62.5)	3 (37.5)	

Employment status				0.268

Employed	40	28 (70.0)	12 (30.0)	

Self employed	174	113 (64.9)	61 (35.1)	

Unemployed	174	130 (74.7)	44 (25.3)	

Not stated	10	7 (70.0)	3 (30.0)	

**Table 2:** Frequency of traditional medicines used during pregnancy and at childbirth (*Other herbal medicines: *Gymnosporia senegalensis* (n=2), *Ipomoea batatas* (n=20), *Dichrostachys cinerea* (n=1), Aloe vera (n=1), lemons (n=1), *Bidens pilosa* (n=1), *Cussonia arborea* (n=1), *Lannea discolour* (n=1), *Erythrina abyssinica* (n=1); **Other traditional medicines: Soil from an anthill (n=3), soil from a rabbit hole (n=3), eggs (n=2), cow dung (n=1), rabbit dung (n=1), vaseline (n=1).

Name of medicine	Common name	Period of use	Frequency (N, %)	Dosage and dosage frequency	Cost (US$)	Location
Soil from a mole hill	Ivhu renhuta	From 3^rd^ trimester to facilitate birth	79 (28.4%)	One fistful in 500 ml of water, drink when thirsty	Free	Homestead/field
Elephant dung	Ndove yenzou	From 3^rd^ trimester to facilitate birth and to prevent tears	59 (21.2%)	One small piece in a cup, drink twice daily	0.5–1	Mbare market
Holy water (from apostolic faith sect)	Mvura yemuteuro	From 1^st^ trimester to protect the child, to ensure a safe delivery	52 (18.7%)	Drink one cup three times daily OR put a cup in bathing water and bath twice daily for 3 days each month	Free	Apostolic sect church
*Fadogia ancylantha*	Makoni tea	From 2^nd^ trimester to facilitate childbirth	37 (13.3%)	One teaspoon in a cup of boiling water, drink once daily	Free	Forest
Cooking oil	-	From 2^nd^ trimester to relax the muscles	33 (11.9%)	One tablespoon, once daily	1.5	Store
Laundry soap (green or blue)	-	From 2^nd^ trimester to widen the birth canal	17 (6.1%)	Lather using hands and insert the hand into the vagina once daily	1	Store
*Abelmoschus esculentus*	Derere	From 3^rd^ trimester to widen the birth canal	10 (3.6%)	Soak overnight, lather hand with water and insert into the vagina twice daily	Free/0.50	Garden/market
*Pinus roxburghii*	Mukirisimasi tree	From 3^rd^ trimester to facilitate childbirth	9 (3.2%)	Dried bark is added to porridge in the morning	Free	Forest
*Dicerocaryum* *zanguebarium*	Ruredzo	From 2^nd^ trimester to widen the birth canal	8 (2.9%)	Soak overnight, lather hands and insert the hand into the vagina once daily	Free	Forest
Muddy nest of a Mud dauber	Chimba chezingizi	At delivery to speed up labour	8 (2.9%)	Soak in a cup, drink when labour starts	Free	Homestead
*Pouzolzia mixta*	Munhanzva	From 3^rd^ trimester to facilitate childbirth	5 (1.8%)	Drink one tablespoon in a cup of water daily	Free	Forest
*Ricinus communis L.*	Mupfuta	At delivery to prevent tears	5 (1.8%)	Seeds are crushed 2 tablespoons of extracted oil is drank	Free	Forest
*Other herbal medicines	-	During pregnancy	13 (4.7%)	Use varies	Free	Forest/homestead
**Other traditional medicines	-	During pregnancy	14 (5%)	Use varies	Free	Forest/homestead

**Table 3:** Reported adverse event profile.

Side effect	Frequency (n)	Frequency (%)
Itchiness	7	27.2
Bitter taste	4	15.4
Bleeding	3	11.5
Irritation	2	7.7
Diarrhoea	2	7.7
Foul vaginal smell	2	7.7
Rash	1	3.8
Vomiting	1	3.8
Still birth	1	3.8
Weak cervix	1	3.8
Vaginal tears	1	3.8
Bloating	1	3.8
Total	26	100
